# A Single Organic Fluorescent Probe for the Discrimination of Dual Spontaneous ROS in Living Organisms: Theoretical Approach

**DOI:** 10.3390/molecules28196983

**Published:** 2023-10-09

**Authors:** Liang Fu, He Huang, Zhongfu Zuo, Yongjin Peng

**Affiliations:** Modern Industry School of Health Management, Jinzhou Medical University, Jinzhou 121001, China

**Keywords:** fluorescent probe, double-lock, sensing mechanism, electronic structure, electron excitation

## Abstract

Single-organic-molecule fluorescent probes with double-lock or even multi-lock response modes have attracted the attention of a wide range of researchers. The number of corresponding reports has rapidly increased in recent years. The effective application of the multi-lock response mode single-molecule fluorescent probe has improved the comprehensive understanding of the related targets’ functions or influences in pathologic processes. Building a highly efficient functional single-molecule fluorescent probe would benefit the diagnosis and treatment of corresponding diseases. Here, we conducted a theoretical analysis of the synthesizing and sensing mechanism of this kind of functional single-molecule fluorescent probe, thereby guiding the design and building of new efficient probes. In this work, we discuss in detail the electronic structure, electron excitation, and fluorescent character of a recently developed single-molecule fluorescent probe, which could achieve the discrimination and profiling of spontaneous reactive oxygen species (ROS, •OH, and HClO) simultaneously. The theoretical results provide insights that will help develop new tools for fluorescent diagnosis in biological and medical fields.

## 1. Introduction

The variation in the properties of a fluorescent probe (such as intensity, wavelength, and half-life) after reacting with its target can be used to achieve specificity for the corresponding target. This technique can be dated back to the 1960s [1,2,3]. F. Goppelsroder built a fluorescent sensor that was used for the detection of Al^3+^ through fluorescence enhanced after chelate formation from mulberry pigment dye with the Al^3+^ ligand. Afterwards, a large number of fluorescent probes for the detection of different kinds of targets were developed [4,5,6,7,8,9,10,11,12,13]. Nowadays, fluorescent probes based on small organic molecules have been widely expanded in food detection, environmental monitoring, clinical examination, and many other fields. In particular, with the rapid development of microscopic imaging technology, fluorescent probes can effectively be used in the in situ and non-invasive imaging and tracking of organisms [10,14,15,16,17,18,19,20,21,22]. This kind of inherent non-invasive imaging analysis ability has an incomparable advantage over other classic biological imaging technologies. Combined with double photon confocal imaging technology, such fluorescent probes can effectively improve the deep monitoring level of related physiological and pathological processes in living organisms with high resolution [23,24,25].

With the early, in-depth research addressing complex biological diseases, it was found that multi-markers (several markers related to one kind of disease) existed in the diseased tissue and organelles [26,27]. So, it was expected that fluorescent probes that could respond to multiple targets would be useful for the detection and monitoring of the early development processes of biological diseases. A pathological process normally needs a variety of indicators to be determined. The synchronous real-time imaging of multiple biomarkers can promote understanding of the correlation between biomarkers and diseases. Obviously, a single target response of a fluorescent probe lacks the required accuracy for understanding disease development, which remains to be improved. Fluorescent probes with double-lock or multi-lock response modes have been popular in recent years [28,29,30,31,32,33,34,35,36,37,38,39]. In this strategy, the fluorescent probe may contain two or more response sites and thus be multi-stimulated. It can also contain a response to one site, and the second response site could be exposed after a specific chemical reaction.

A single-molecule fluorescent probe with a multi-lock response mode could overcome many shortcomings of a probe with a single response site. Not only could the cross interference between signals be significantly reduced, but also the spatial resolution of the image could be highly improved. The real-time synchronization imaging of multiple biomarkers through the single-molecule fluorescent probe could clarify the relationship between the basic research biomarkers and eventually realize the precision detection and monitoring of the development process of the diseases.

The incomplete reduction of molecular oxygen under external stimulations or injuries in the cellular mitochondria of various organs and tissues of living organisms could produce lots of reactive oxygen species. 

Biological anomalies such as cell aging, DNA/protein mutations, and various diseases could be caused by the excessive generation of reactive oxygen species. Each individual reactive oxygen species, such as hypochlorous acid (HClO), hydroxyl radicals (•OH), and hydrogen peroxide (H_2_O_2_), has unique functions that remain ambiguous [40,41,42,43,44,45,46]. Accordingly, a powerful tool to reveal their functions and correlations with biological processes is greatly demanded by researchers and practitioners in the medical field.

**Figure 1:**
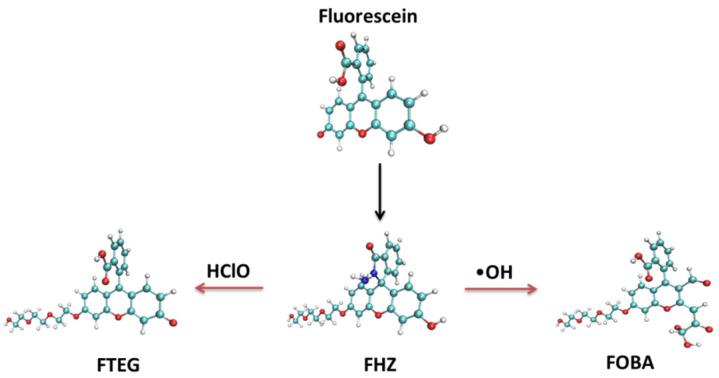
The synthetic process of the probe FHZ and its reaction with •OH and HClO.

Large invasive effects and discrepant biological distributions of multiple probes hinder the effective simultaneous detections of several reactive oxygen species. A single fluorescent probe that can respond to several reactive oxygen species with distinguishable fluorescent signals would be very helpful for studying the correlation between reactive oxygen species and the life process. Such a single fluorescent probe, 6-triethyleneglycol substituted fluorescein hydrazide (FHZ), which could obtain the effective discrimination and profiling of hydroxyl radicals (•OH) and hypochlorous acid (HClO) in living organisms, was reported by Zhang et al. recently [47]. The two reaction sites of the probe FHZ corresponded to •OH and HClO simultaneously, resulting in cyan (486 nm) and green (520 nm) emissions, respectively. An additional five-membered heterocyclic ring and a lateral triethyleneglycol chain were chemically grafted to fluorescein to construct the probe FHZ which was non-fluorescent with dual reactive sites to •OH and HClO, respectively. The synthetic process of the probe FHZ and its reaction with •OH and HClO are illustrated simply in Figure 1. ChemDraw figures of the four molecular structures are given in the supporting information (Figure S1). The successful application of the probe FHZ in different living organisms shows the versatility of this fluorescent probe and its potential as a powerful tool in investigations of reactive oxygen species in living systems.

In this work, the sensing mechanism and spectrum character of probe FHZ were studied in detail under the quantum chemistry method. The electronic structures, reaction sites, and ring conjugation properties of the fluorescent probes FHZ, FTEG (FHZ reacted with HClO), and FOBA (FHZ reacted with •OH) were theoretically analyzed to gain an in-depth understanding of the principle of detection on reactive oxygen species (HClO and •OH) with the fluorescent probe FHZ. These theoretical results could inspire the research community to design and synthesize highly efficient fluorescent probes with multi-response to biomarkers, which could be widely used in biological and medical fields.

## 2. Results

### 2.1. Electronic Structures

**Figure 2:**
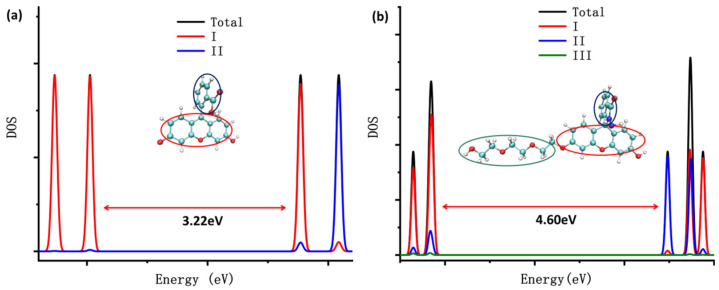

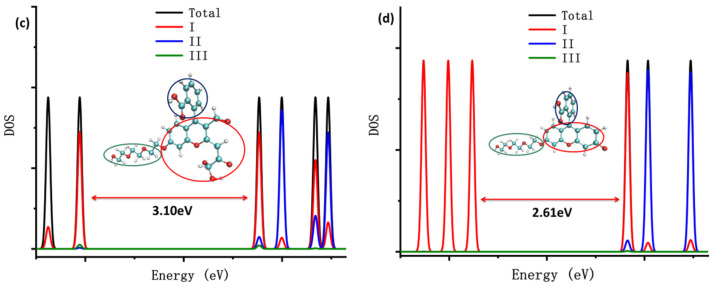
The density of states (DOS) of (**a**) fluorescein, (**b**) FHZ, (**c**) FOBA, and (**d**) FTEG. (black: total dos; red: xanthene part; blue: 2-Carboxyphenyl part; and green: Triethylene Glycol methyl ether part).

The density of states (DOS) of the probes fluorescein, FHZ, FOBA, and FTEG were obtained through Multiwfn 3.8(dev) and are illustrated in Figure 2. Figure 2 clearly shows that the highest occupied molecular orbits (HOMOs) of the four probes were mainly contributed from part I (xanthene part, red circle in Figure 2) in the molecule. The lowest unoccupied molecular orbits (LUMOs) of the probes fluorescein, FTEG, and FOBA were also mainly contributed from part I; otherwise, the LUMO of probe FHZ was contributed from part II (2-Carboxyphenyl part, blue circle in Figure 2) in the molecule. Part III (Triethylene Glycol methyl ether part, green circle in Figure 2) in the probe molecules FHZ, FTEG, and FOBA makes a negligible contribution to the molecule orbits near the HOMO and LUMO energy level. The different electronic structure of probe FHZ was probably due to the five-membered heterocyclic ring, which contains the N atoms. Yet it was the five-membered heterocyclic ring, which contains the N atoms and provides the reaction sites, that corresponded to the reactive oxygen species (HClO and •OH). 

**Figure 3:**
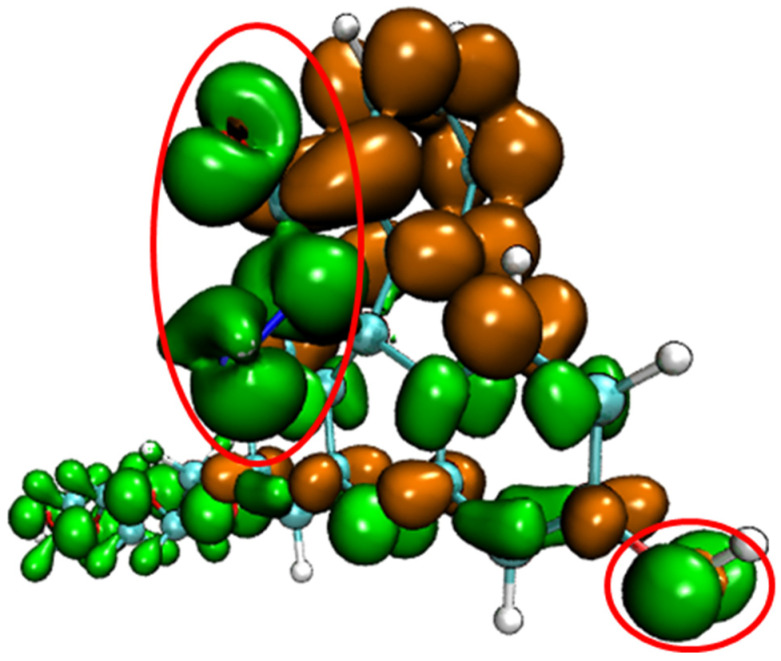
The dual descriptor of probe FHZ (isovalue = +/−0.0005, orange: positive, green: negative) two red circles indicated the five-membered heterocyclic ring and hydroxyl parts of the probe FHZ.

Conceptual density functional theory (CDFT), also known as density functional reactivity theory (DFRT), which was initially developed by Parr, is an important component in the field of quantum chemistry and wave function analysis [48,49]. It is especially useful in predicting and explaining the reaction activity and reaction site of chemical structures. Fukui function and dual descriptor are very popular methods for predicting reaction sites defined under the conceptual density functional theory framework [50]. The dual descriptor of probe FHZ was obtained through Multiwfn 3.8(dev) analysis based on the ORCA output results and is illustrated in Figure 3. 

**Figure 4:**
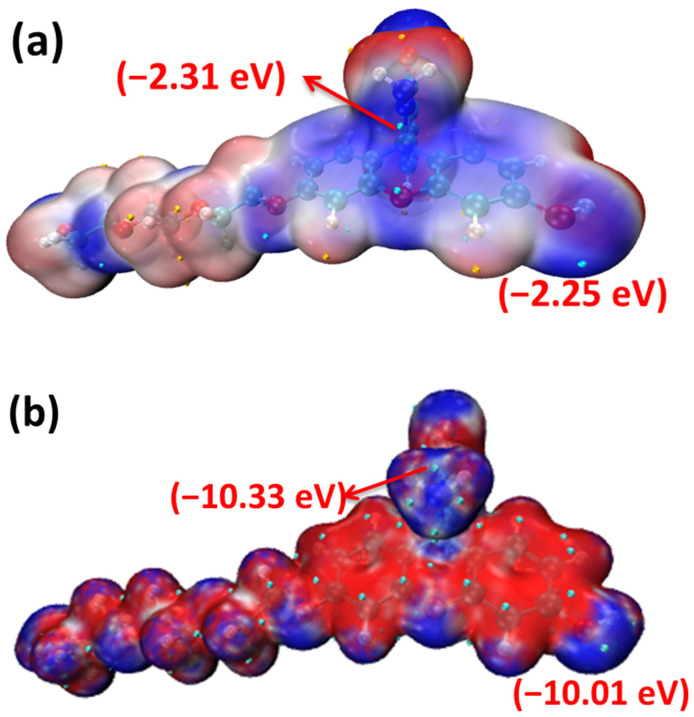
The electrostatic potential (**a**) and local electron affinity (**b**) of the probe FHZ red: positive, purple: negative.

Figure 3 clearly shows that the five-membered heterocyclic ring and hydroxyl parts of the probe FHZ (red circle in Figure 3) were the electrophilic reaction sites. The reaction of FHZ with reactive oxygen species (HClO and •OH) to FTEG and FOBA was consistent with the theoretical analysis from Figure 3. Moreover, the electrostatic potential (ESP) and local electron affinity (LEA) of the probe FHZ were both analyzed through Multiwfn 3.8(dev), and the results are illustrated in Figure 4. 

The distribution and extremum points of ESP and LEA shown in the probe FHZ structure clearly indicated the potential reaction sites located near the five-membered heterocyclic ring and hydroxyl parts, which was consistent with the dual descriptor theoretical analysis and the experimental results. 

**Figure 5:**
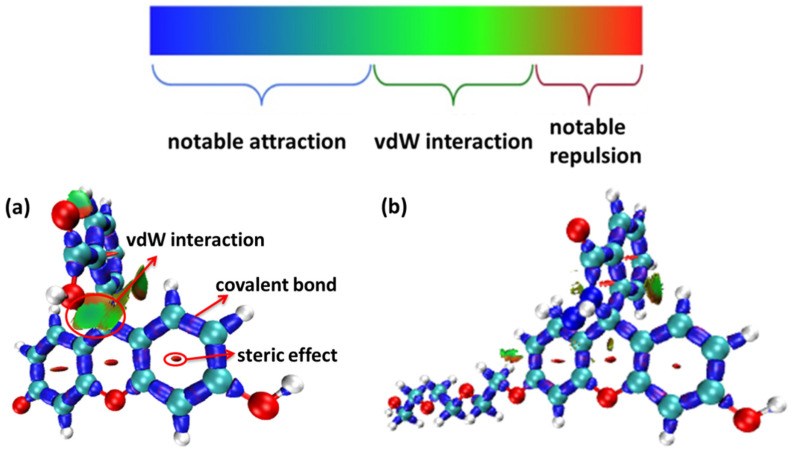

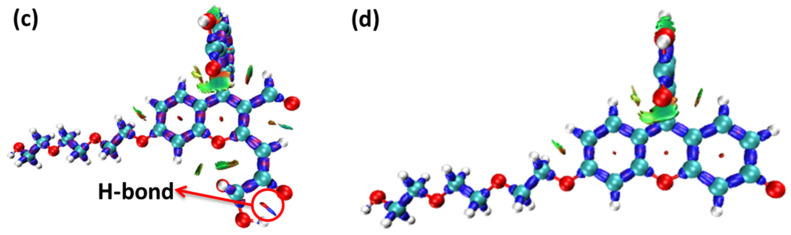
The interaction region indicator (IRI) function of (**a**) fluorescein, (**b**) FHZ, (**c**) FOBA, and (**d**) FTEG (isovalue = 0.05).

**Figure 6:**
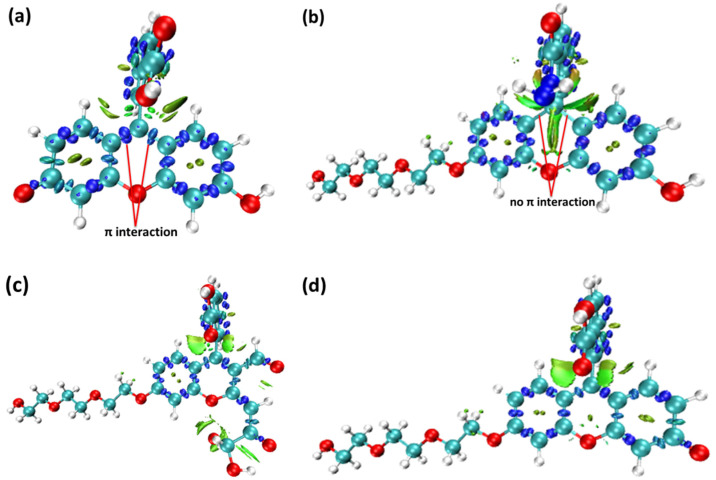
The interaction region indicator of π electrons (IRI-π) function of (**a**) fluorescein, (**b**) FHZ, (**c**) FOBA, and (**d**) FTEG (isovalue = 0.05).

To further uncover the difference in the electronic structures of fluorescein, probes FHZ, FOBA, and FTEG, the interaction region indicator (IRI) function and IRI-π, which was only considered as the interaction between the π electrons in the molecule of the four probes, were obtained through Multiwfn 3.8(dev) analysis based on the ORCA output results and are illustrated in Figure 5 and Figure 6, respectively. 

**Figure 7:**
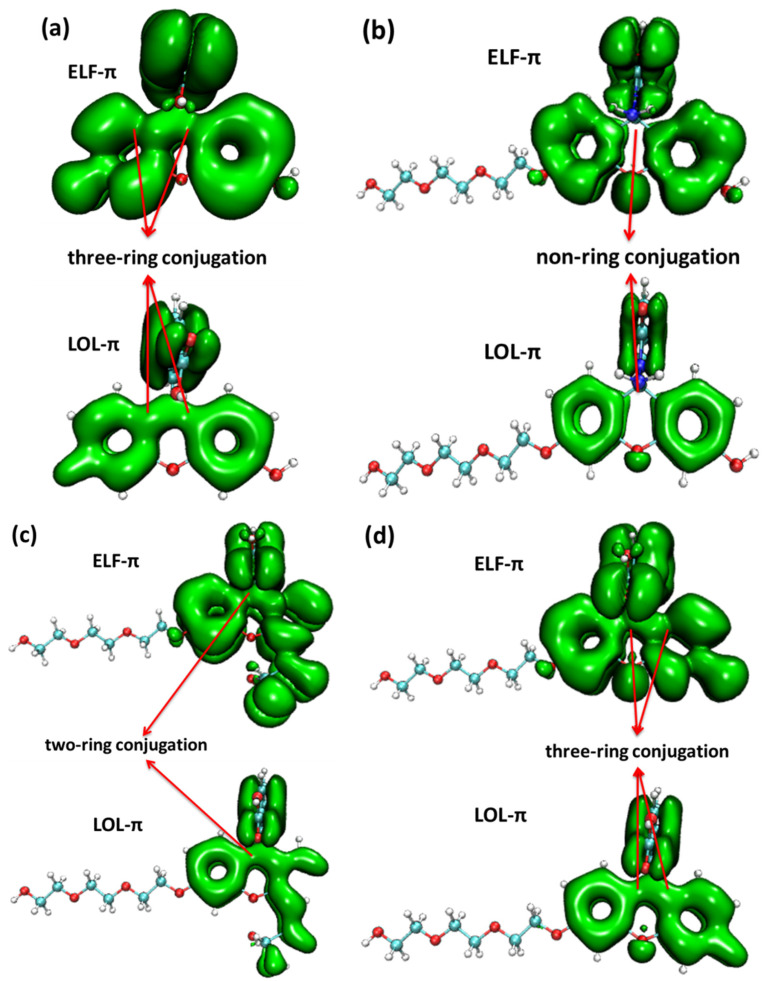
The electron localization function (ELF) and localized orbital locator (LOL) of π electrons in (**a**) fluorescein, (**b**) FHZ, (**c**) FOBA, and (**d**) FTEG (isovalue = 0.05).

From the IRI function of the four probes (Figure 5), it can be clearly seen that the vdW interaction, covalent bond, and steric effect coexisted in the probe molecules, including the H-bond in the molecule FOBA. Differences can hardly be found among the four probe molecules. However, when only the interaction between π electrons was considered (IRI-π, as shown in Figure 6), the difference in the probe FHZ compared with the other three molecules was clearly shown. The π-interaction between the top two carbon atoms in the middle six-membered heterocyclic ring clearly indicated the three-ring (two-ring) conjugation in the probes fluorescein and FTEG (FOBA). Whereas there was no π-interaction between the top two carbon atoms in the down-middle six-membered heterocyclic ring of the probe molecule FHZ, indicating there was non-ring conjugation in this probe molecule, which may lead to its non-fluorescence property. To further confirm the conjugation and aromatic properties of the four probe molecules, the electron localization function (ELF) and localized orbital locator (LOL) of π electrons in the four probe molecules were analyzed through Multiwfn 3.8(dev) and are shown in Figure 7.

From the map of the isovalue of ELF-π and LOL-π within the four probe molecules, it could be clearly shown that the break of the ELF-π and LOL-π in the middle six-membered heterocyclic ring of probe molecule FHZ indicated the non-ring conjugation and low aromaticity in this molecule. Whereas the continuous nature of the ELF-π and LOL-π between the six-membered heterocyclic rings in the other three probe molecules (fluorescein, FTEG, and FOBA) indicated the three (two)-ring conjugation and high aromaticity in these probe molecules. This conclusion was consistent with the results obtained from the π electrons interaction analysis mentioned above.

### 2.2. Fluorescent Properties

**Figure 8:**
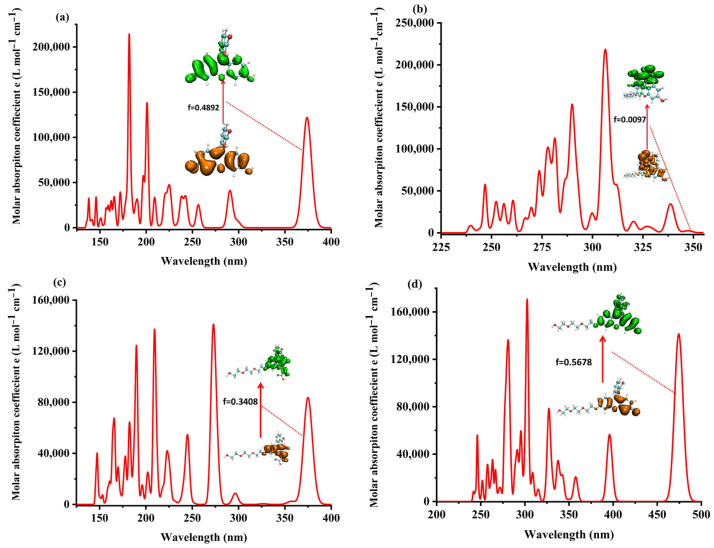
The theoretical ultraviolet absorption spectrum of (**a**) fluorescein, (**b**) FHZ, (**c**) FOBA, and (**d**) FTEG.

The time-dependent density functional theory (TDDFT) was used for studying the electronic excited states of the four probe molecules. The theoretical ultraviolet absorption spectrum and the corresponding electron excitation in the process from ground state S_0_ to the lowest singlet excited state S_1_ of the four probe molecules are shown in Figure 8.

The analysis of the electron excitation from S_0_ to S_1_ showed that it was mainly the electron excited from the HOMO to LUMO with relatively significant oscillator strength in fluorescein, FTEG, and FOBA molecules. In the probe molecule FHZ, the electron was excited from the HOMO–−1 to LUMO + 1, HOMO–−2 to LUMO + 2, and HOMO to LUMO in the process from S_0_ to S_1_ with only relatively negligible oscillator strength, which was correlated with its non-fluorescence property. The details of the theoretical electron excitation and emission processes and the experimental fluorescence of the four probe molecules are summarized in Tables S1 and S2, respectively.

**Figure 9:**
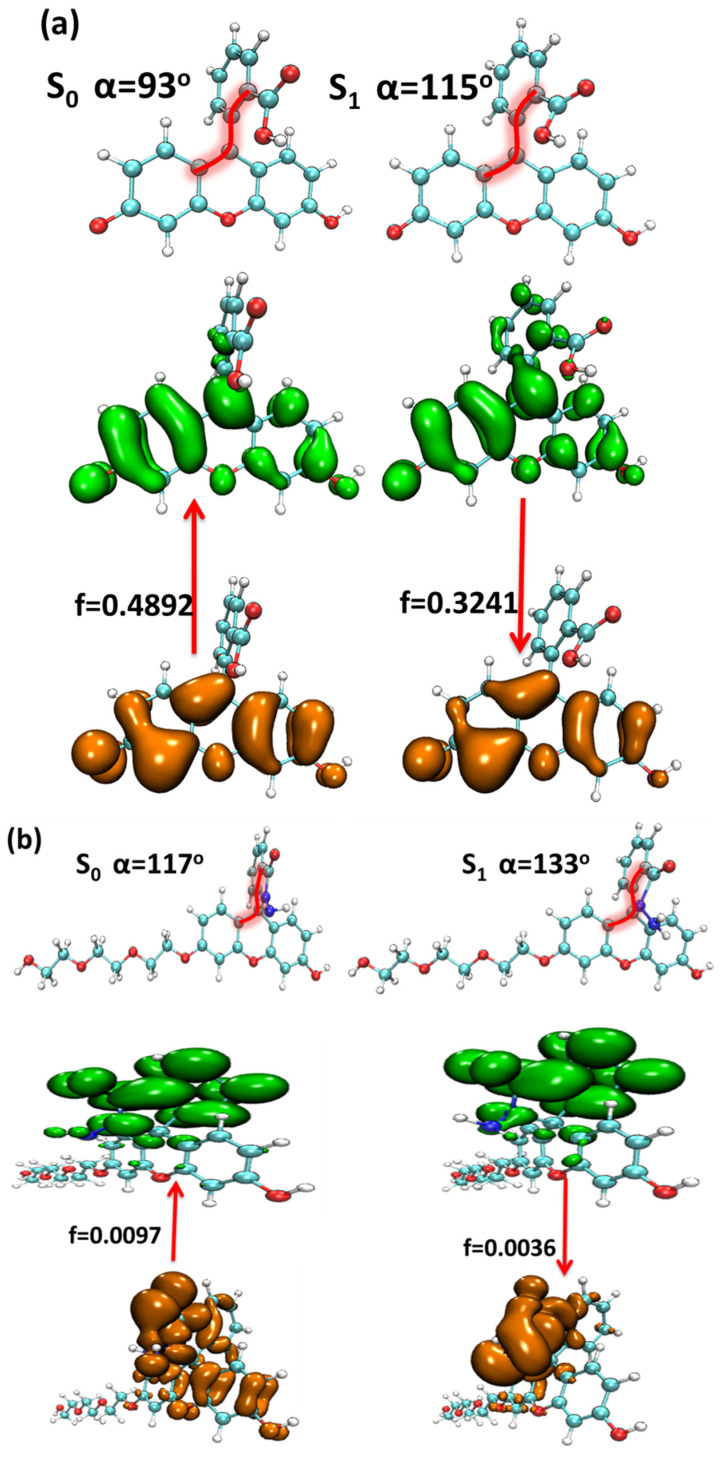

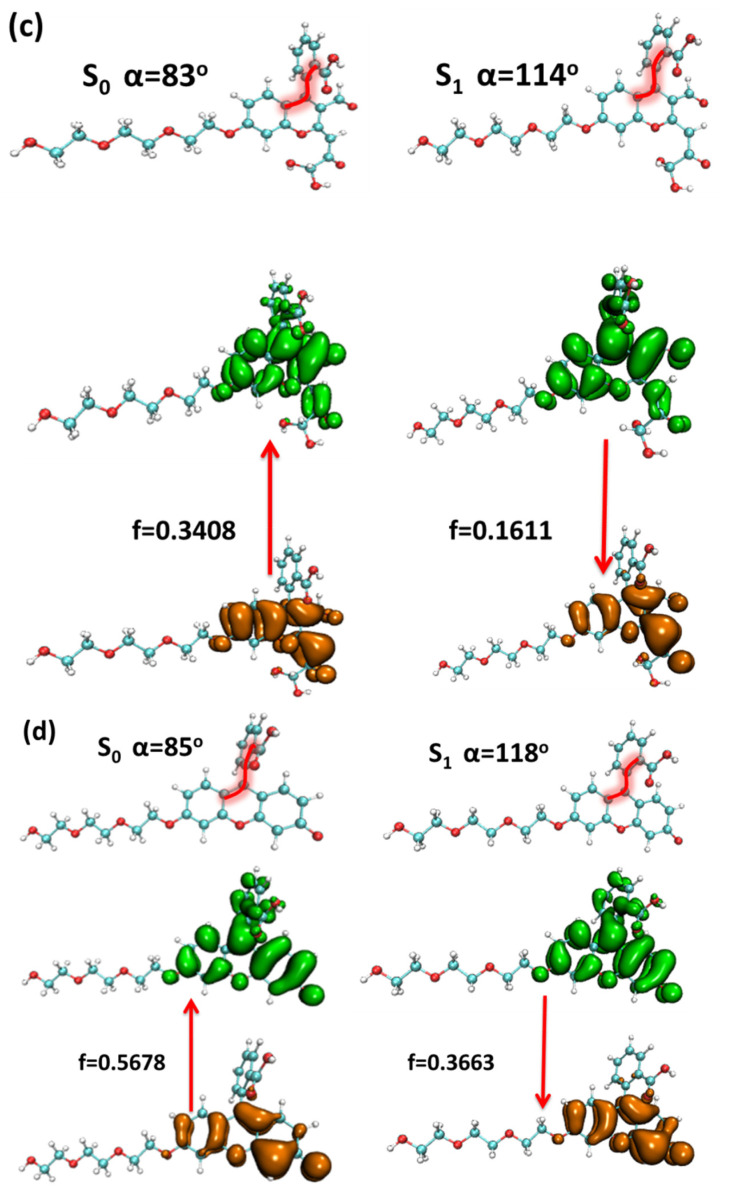
The structure variation and electron excitation and emission processes between the S_0_ and S_1_ of (**a**) fluorescein, (**b**) FHZ, (**c**) FOBA, and (**d**) FTEG (isovalue = 0.05, orange: hole; green: electron).

The elaborate study on the structure variation and electron excitation and emission processes between S_0_ and S_1_ of the four probe molecules is shown in Figure 9.

**Figure 10:**
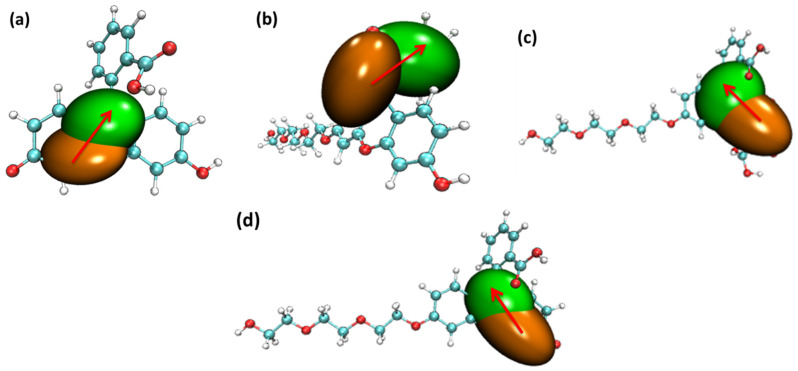
The center of the hole and electron’s distribution for the four probe molecules from S_0_ to the S_1_ state: (**a**) fluorescein, (**b**) FHZ, (**c**) FOBA, and (**d**) FTEG (isovalue = 0.002 orange: hole; green: electron, red arrows indicated the direction of electron transfer).

**Figure 11:**
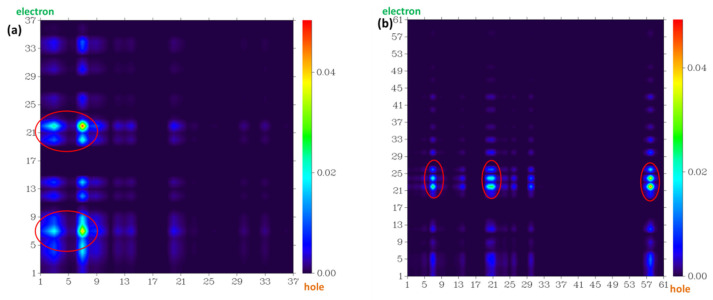

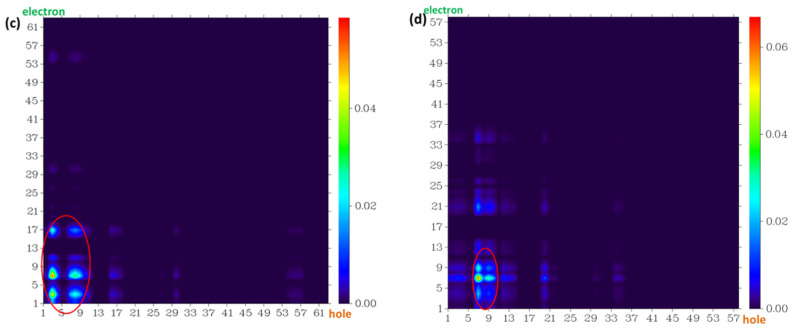
The atom–atom transfer heat map for the four probe molecules from S0 to the S1 state: (**a**) fluorescein, (**b**) FHZ, (**c**) FOBA, and (**d**) FTEG. The contribution to the hole (electron) from all of the atoms was normalized to unity (red circle indicated the atoms with big contribution).

The structural difference between the S_0_ and S_1_ of the four probe molecules was mainly the variation of the dihedral angle α between the down-middle six-membered heterocyclic ring and the up-benzene ring, as shown in Figure 9. The dihedral angle α changed from nearly 90° (the down-middle six-membered heterocyclic ring was perpendicular to the up-benzene ring) to more than 110° with a little twisted excited structure in the probes fluorescein, FTEG, and FOBA. Whereas there was a five-membered heterocyclic ring located between the down-middle six-membered heterocyclic ring and up-benzene ring in the probe molecule FHZ, the dihedral angle α was already 117° in S_0_, which was deviated from 90°. From S_0_ to the S_1_ state, the dihedral angle α was simply changed from 117° to 133° with a smaller change compared with the other three probe molecules. There was a significant oscillator strength larger than 0.3 between the S_0_ and S_1_ states of the three probe molecules (fluorescein, FTEG, and FOBA), which indicated their corresponding strong fluorescence. The non-fluorescence of probe molecule FHZ could be attributed to the negligible oscillator strength being smaller than 0.01 between the S_0_ and S_1_ states of the probe molecule. Another obviously different character of electron excitation from S_0_ to the S_1_ state between the probe molecule FHZ and the other three probe molecules (fluorescein, FTEG, and FOBA) was that there was basically the local electron excitation in the pyran rings of the latter three probe molecules, whereas there was electron transfer between the five-membered heterocyclic ring and the adjacent benzene ring in the probe FHZ. The center of the hole and electron distribution and the atom–atom transfer heat map of the four probe molecules from S_0_ to the S_1_ state clearly delineated the different characters between FHZ and the other three probe molecules, as shown in Figure 10 and Figure 11, respectively. The number of atoms in the four probe molecules is referenced in Figure S2.

The radiative lifetime $\tau_{r}$ and fluorescence quantum yield ∅ of the four molecules were obtained through FCclasses 3.0.1 [51,52]. The corresponding theoretical and experimental results are summarized in Table 1.

## 3. Materials and Methods 

The stable ground state geometric structures of the four probes (fluorescein, FHZ, FETG, and FOBA) were searched under the combination of PBE0/def2-TZVPD with D3 dispersion and geometrical counterpoise (gCP) correction to remove artificial overbinding effects from Basis Set Superposition Error (BSSE) through the ORCA 5.0.1 program [53,54,55,56,57].

Non-imaginary frequency was found in the vibrational analysis on the stable geometric structure, which confirmed the stability of the results.

The electron excitation and fluorescent properties of the four probe molecules were analyzed through the time-dependent density functional theory (TDDFT) method, which was performed under CAM-B3LYP/def2-TZVPD. This functional and basis set combination was confirmed to be appropriate for studying the excited states of single organic molecules [58,59,60,61]. TDDFT calculation under functional M06-2X and PBE0 was also conducted to be compared with the results under CAM-B3LYP. Except for a small quantitative difference, similar qualitative results were obtained from these three XC functionals, which did not change the conclusion. The corresponding analyses were finished using Multiwfn 3.8(dev) code [62], and some of the figures were delineated through VMD 1.9.3 software [63]. The orange and green isosurfaces in the figures represent the hole and electron distribution, respectively.

## 4. Conclusions

The sensing mechanism of the single-organic-molecule fluorescent probe FHZ, which could achieve the discrimination of dual ROS (•OH and HClO) in living organisms, was studied thoroughly under quantum mechanical methods. The dual descriptor, ESP, and LEA of the probe FHZ all indicated that the response sites to ROS (•OH and HClO) were the five-membered heterocyclic ring and hydroxyl parts in the probe molecule. The electronic structures’ difference in the probes was the origin of their different fluorescent characters, which led to the applicability for the sensing of ROS through fluorescence detection. The non-ring conjugation in the probe FHZ may be related to its non-fluorescence property. The different wavelengths (color) of the fluorescence of FTEG (FHZ reacted with HClO) and FOBA (FHZ reacted with •OH) realized the detection the •OH and HClO through the probe FHZ, respectively. All of these theoretical results could be beneficial for paving the route for designing multi-lock mode fluorescent probes applicable in biological fields with high efficiency.

## Figures and Tables

**Table 1:** Calculated radiative lifetime $\tau_{r}$ (ns) and radiative and non-radiative decay rate $k_{r}$ and $k_{nr}$ (in $\left[10^{-7}s^{-1}\right]$), respectively. Calculated and measured fluorescence quantum yield ∅ and $\emptyset^{exp}$ [47].

	$\tau_{r}$	$k_{r}$	$k_{nr}$	∅	$\emptyset^{exp}$
Fluorescein	21.69	4.61	6.37	0.42	----
FHZ	500.01	0.20	21.04	0.01	0.01
FOBA	23.26	4.31	6.62	0.39	0.42
FTEG	11.90	8.40	17.05	0.33	0.34

## Data Availability

Corresponding data could be obtained on request through author’s email.

## Supplementary Materials

The following supporting information can be downloaded at: https://www.mdpi.com/article/10.3390/molecules28196983/s1. Figure S1: the chemdraw structure of probe molecules fluorescein, FHZ, FOBA and FTEG; Figure S2: the stable structure of probe molecules (a) fluorescein, (b) FHZ, (c) FOBA and (d) FTEG; Table S1: The main electron excitation processes in the probe molecules. Table S2: The main electron emission processes in the probe molecule.
