# Supplementary material for: A Single Organic Fluorescent Probe for the Discrimination of Dual Spontaneous ROS in Living Organisms: Theoretical Approach
Source: Molecules. 2023 Oct 9;28(19):6983. doi: 10.3390/molecules28196983 (PMC10574373; doi:10.3390/molecules28196983)
Supplement: Supplementary file 1 [file molecules-28-06983-s001.zip › molecules-2599055-supplementary.pdf]

A Single Organic Fluorescent Probe for the Discrimination of Dual Spontaneous ROS in Living Organisms: Theoretical Approachs

Liang Fu, He Huang, Zhongfu Zuo and Yongjin Peng *

Modern Industry School of Health Management, Jinzhou Medical University, Jinzhou 121001, China
* Correspondence: hunterpyj2016@163.com

Table S1. The main electron excitation processes in the probe molecules

Compound	Electronic transition[a]	Excitation Energy	Oscillator strengh	Composition[b]	CI[c]
Fluorescein	$S_0 \rightarrow S_1$	3.22eV	0.4892	H → L	0.6814
FHZ	$S_0 \rightarrow S_1$	4.60eV	0.0097	H → L	0.2005
				H-1 → L+1	0.5291
				H-2 → L+2	0.1846
FOBA	$S_0 \rightarrow S_1$	3.10eV	0.3408	H → L	0.6912
FTEG	$S_0 \rightarrow S_1$	2.61eV	0.5678	H → L	0.6769

a Only the excited states with oscillator strength larger than 0.1 were considered. b H stand for HOMO and L stands for LUMO. c Coefficient of the wave function for each excitation was in absolute value.

Table S2. The main electron emission processes in the probe molecule

Compound	Electronic transition[a]	Excitation Energy	Oscillator strengh	Composition[b]	CI[c]
Fluorescein	$S_1 \rightarrow S_0$	2.75eV	0.3241	H → L	0.6901
FHZ	$S_1 \rightarrow S_0$	3.78eV	0.0036	H → L	0.1126
				H-1 → L+1	0.4928
				H-2 → L+2	0.1954
FOBA	$S_1 \rightarrow S_0$	2.61eV	0.1611	H → L	0.6911
FTEG	$S_1 \rightarrow S_0$	2.42eV	0.3663	H → L	0.6756

a,b,c same indication as in Table S1

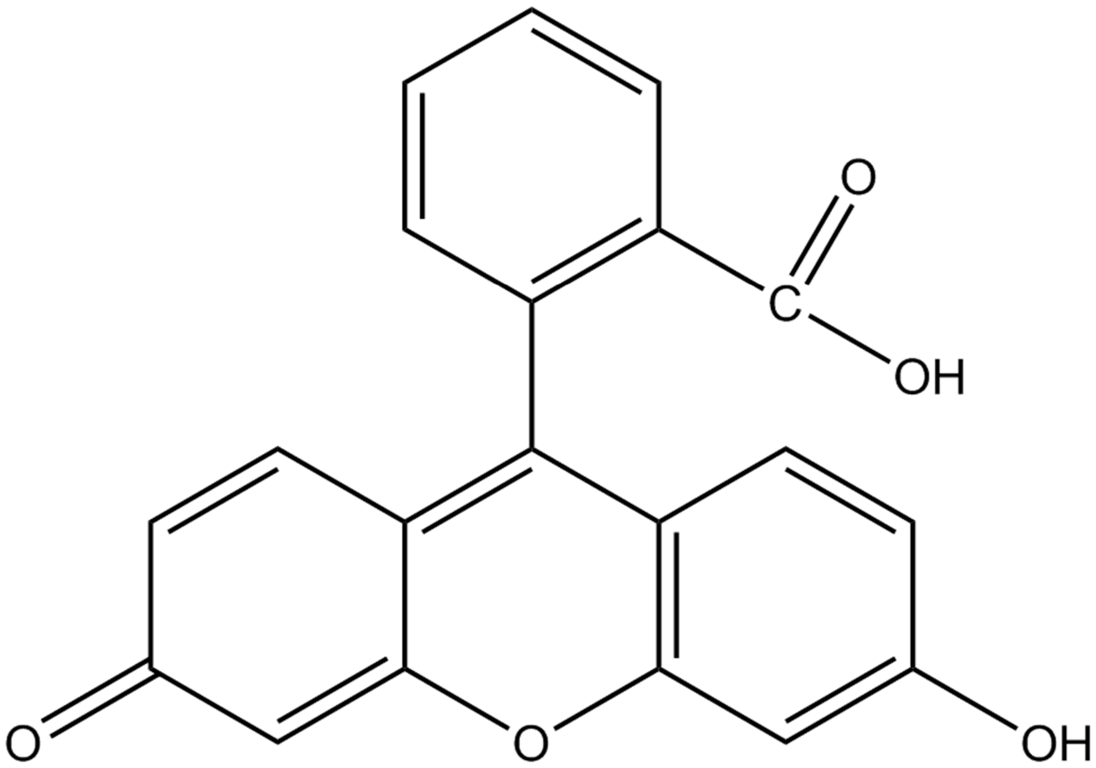

Fluorescein

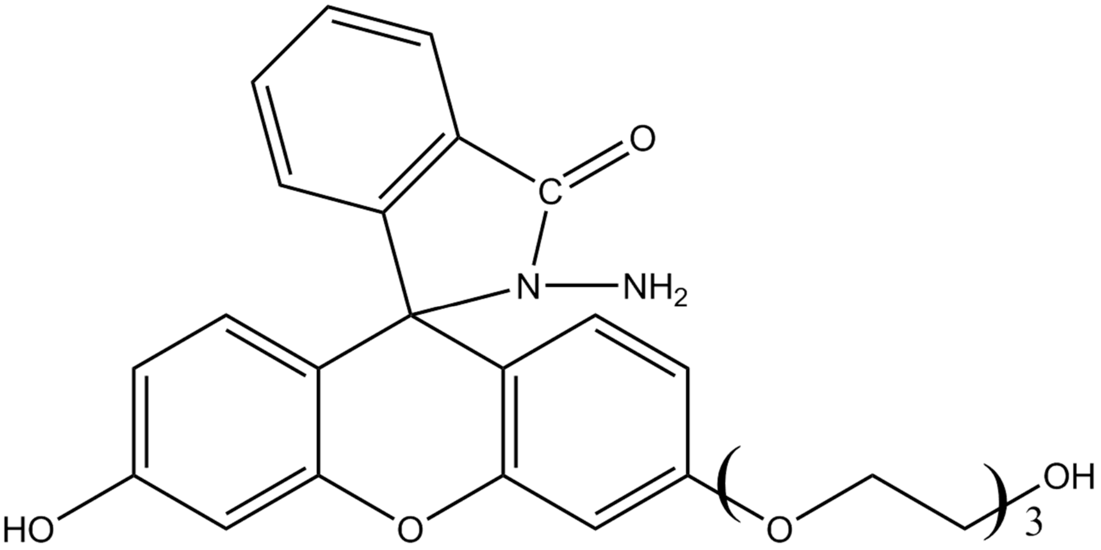

FHZ

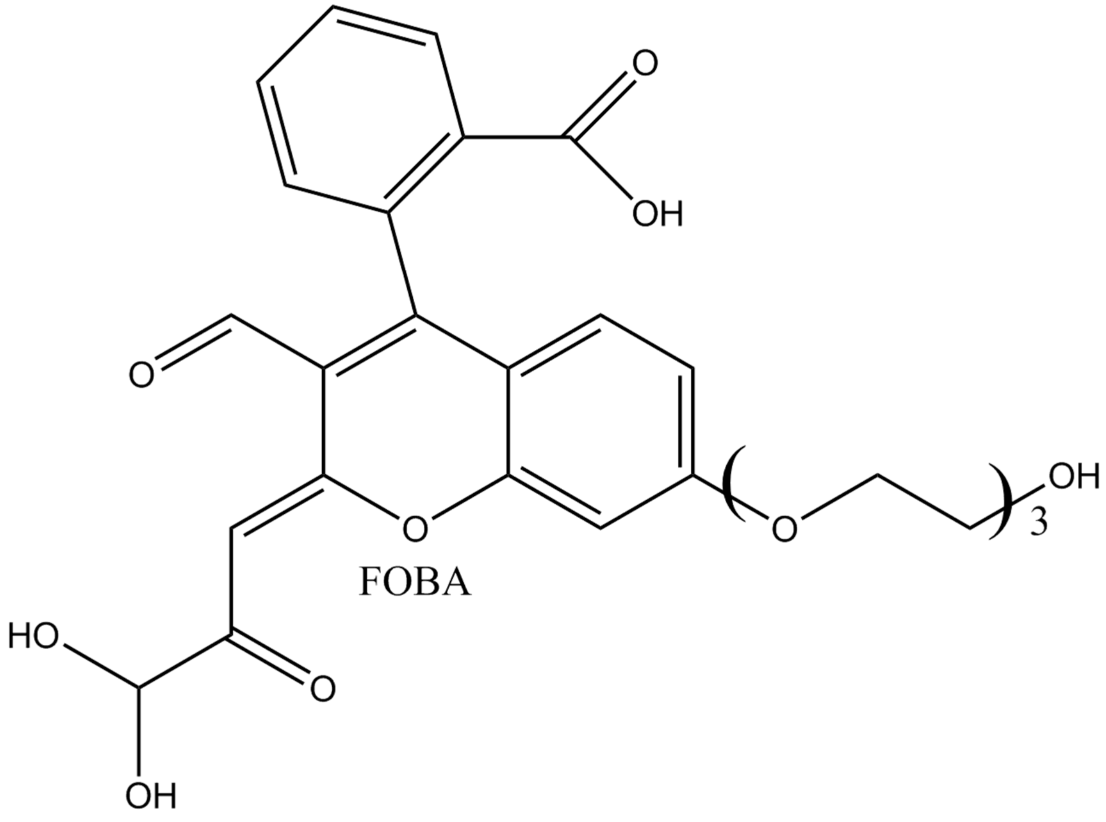

FOBA

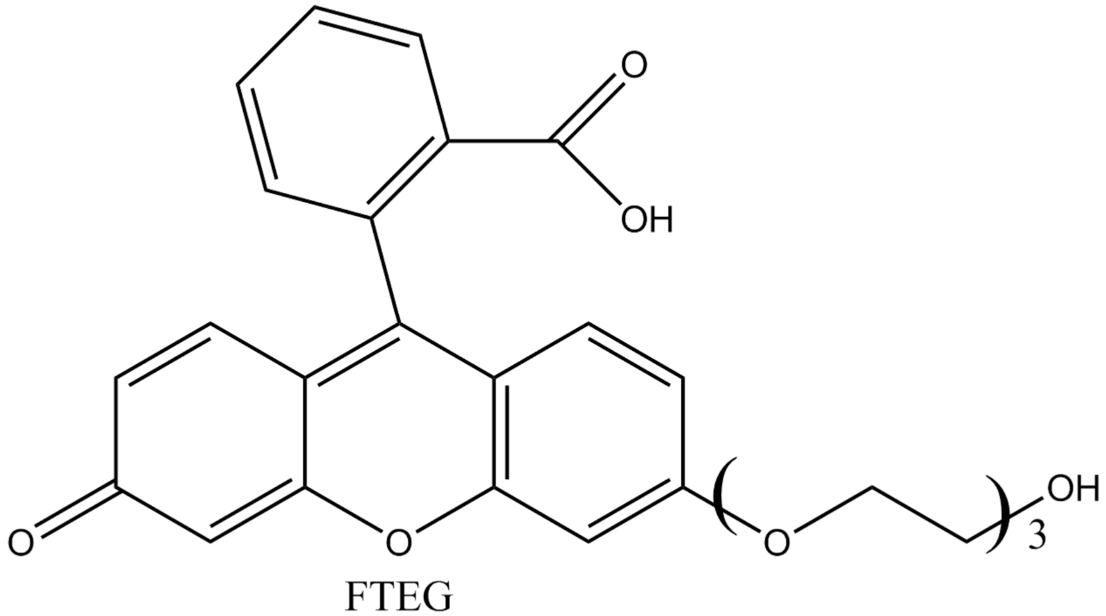

FTEG

Figure S1. The chemdraw structure of probe molecules fluorescein, FHZ, FOBA and FTEG.

(a)

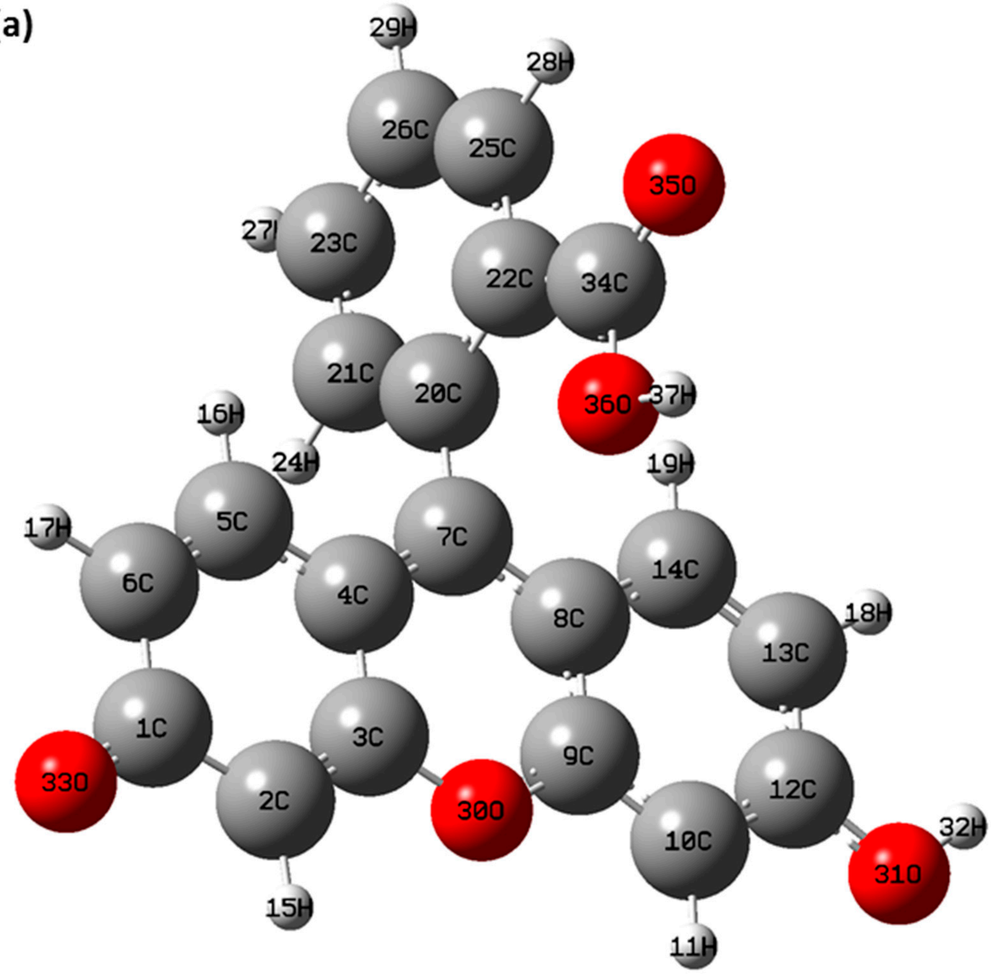
29H
28H
26C
25C
35O
27H
23C
22C
34C
21C
20C
36O
37H
16H
24H
19H
17H
5C
7C
14C
6C
4C
8C
18H
13C
1C
3C
9C
12C
33O
2C
30O
10C
32H
31O
15H
11H

(b)

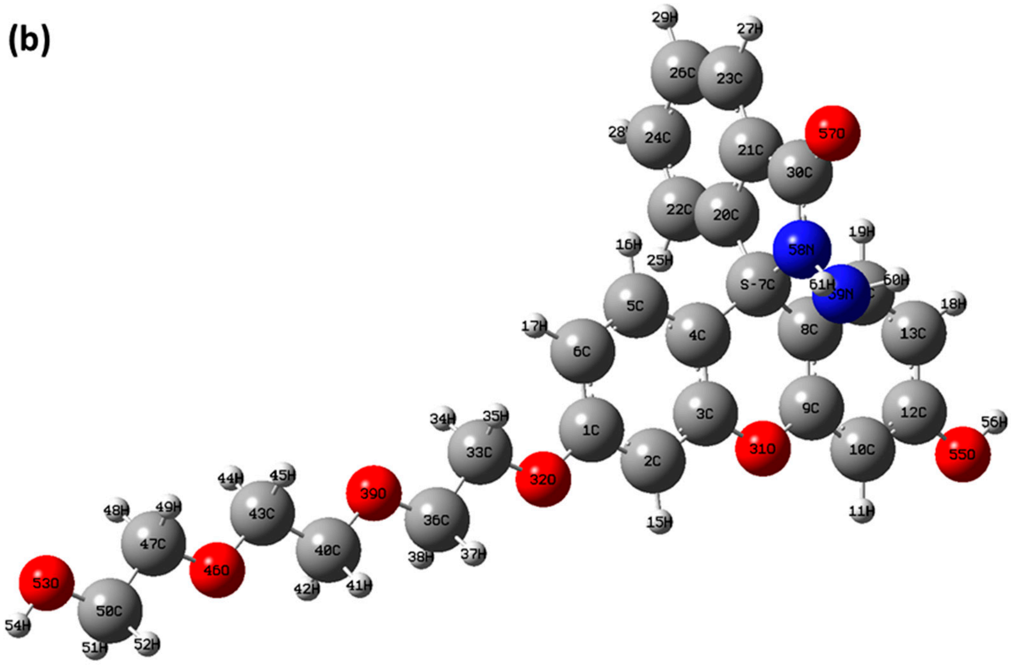
29H
27H
26C
23C
57O
24C
21C
30C
22C
20C
58N
19H
16H
25H
61H
59N
60H
18H
17H
5C
4C
8C
13C
6C
9C
12C
34H
35H
3C
1C
56H
33C
31O
10C
55O
2C
32O
44H
45H
39O
36C
15H
11H
48H
49H
43C
47C
40C
38H
37H
46O
42H
41H
53O
50C
54H
51H
52H

(c)

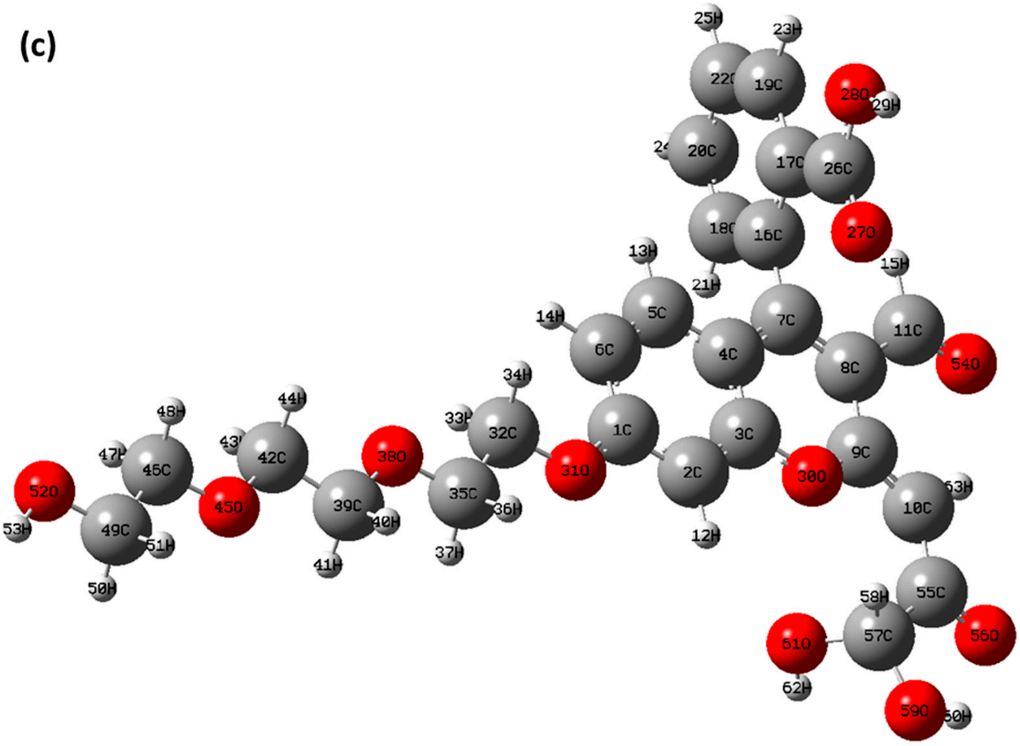

(d)

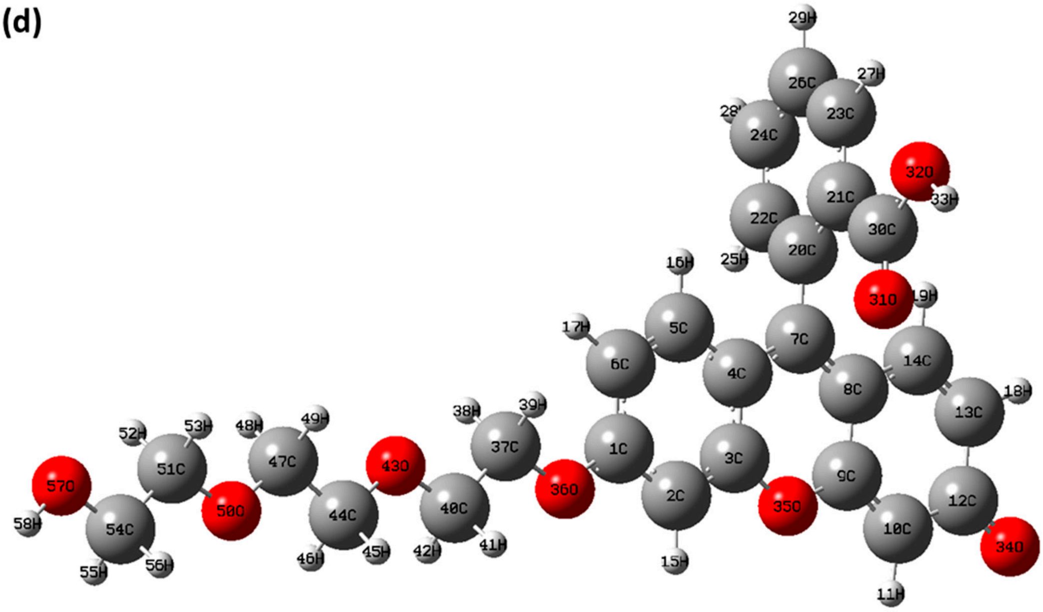

Figure S2. the stable structure of probe molecules (a) fluorescein, (b) FHZ, (c) FOBA and (d) FTEG

Fluorescein

C	−3.98563300	−1.52351000	−3.04867500
C	−2.99230000	−1.67961200	−1.99220700
C	−2.83469800	−0.73683100	−1.02907800
C	−3.62577600	0.47815700	−0.98714200
C	−4.62253200	0.63885100	−2.01984100
C	−4.79515600	−0.29033600	−2.98511700

C	-3.41934000	1.38795100	0.02039100
C	-2.40863900	1.13004400	1.01243600
C	-1.67356000	-0.07112100	0.92195300
C	-0.68859000	-0.39830200	1.84598000
H	-0.13736200	-1.33347400	1.75023100
C	-0.41575900	0.48225200	2.89215300
C	-1.13032200	1.68913200	3.00365300
C	-2.10651400	1.99977900	2.07485600
H	-2.37682500	-2.57975200	-1.99091200
H	-5.23955000	1.53973400	-1.99410400
H	-5.54946600	-0.17296100	-3.76640600
H	-0.91015700	2.37855800	3.82330300
H	-2.65741200	2.93838700	2.16135400
C	-4.17320400	2.66778400	0.07336600
C	-3.74009300	3.69922700	-0.76897000
C	-5.25892400	2.91323100	0.93797200
C	-4.36149800	4.94477200	-0.76285700
H	-2.89718600	3.50976700	-1.43730700
C	-5.87135900	4.17262700	0.93955500
C	-5.43150500	5.18515800	0.09720100
H	-4.00525400	5.73010200	-1.43358500
H	-6.70872800	4.32435700	1.62328300
H	-5.92367600	6.15988900	0.10900500
O	-1.89600800	-0.95160000	-0.06865000
O	0.54053800	0.13255500	3.76588400
H	0.63481800	0.81904000	4.43530000
O	-4.15240200	-2.35289300	-3.93327700
C	-5.84714500	1.91438000	1.87915700
O	-6.70827400	2.18204700	2.67897400
O	-5.34462000	0.68447300	1.75395100
H	-5.79882900	0.13116500	2.40768000

FHZ

C	-3.98563300	-1.52351000	-3.04867500
C	-2.99230000	-1.67961200	-1.99220700
C	-2.83469800	-0.73683100	-1.02907800
C	-3.62577600	0.47815700	-0.98714200
C	-4.62253200	0.63885100	-2.01984100
C	-4.79515600	-0.29033600	-2.98511700
C	-3.41934000	1.38795100	0.02039100
C	-2.40863900	1.13004400	1.01243600
C	-1.67356000	-0.07112100	0.92195300
C	-0.68859000	-0.39830200	1.84598000
H	-0.13736200	-1.33347400	1.75023100

C	-0.41575900	0.48225200	2.89215300
C	-1.13032200	1.68913200	3.00365300
C	-2.10651400	1.99977900	2.07485600
H	-2.37682500	-2.57975200	-1.99091200
H	-5.23955000	1.53973400	-1.99410400
H	-5.54946600	-0.17296100	-3.76640600
H	-0.91015700	2.37855800	3.82330300
H	-2.65741200	2.93838700	2.16135400
C	-4.17320400	2.66778400	0.07336600
C	-3.74009300	3.69922700	-0.76897000
C	-5.25892400	2.91323100	0.93797200
C	-4.36149800	4.94477200	-0.76285700
H	-2.89718600	3.50976700	-1.43730700
C	-5.87135900	4.17262700	0.93955500
C	-5.43150500	5.18515800	0.09720100
H	-4.00525400	5.73010200	-1.43358500
H	-6.70872800	4.32435700	1.62328300
H	-5.92367600	6.15988900	0.10900500
O	-1.89600800	-0.95160000	-0.06865000
O	0.54053800	0.13255500	3.76588400
H	0.63481800	0.81904000	4.43530000
O	-4.15240200	-2.35289300	-3.93327700
C	-5.84714500	1.91438000	1.87915700
O	-6.70827400	2.18204700	2.67897400
O	-5.34462000	0.68447300	1.75395100
H	-5.79882900	0.13116500	2.40768000

FOBA

C	-4.70068700	-3.39073400	1.43915300
C	-3.43438700	-3.26035900	0.86055100
C	-3.04453500	-2.02657200	0.36726400
C	-3.88854500	-0.90068200	0.41746500
C	-5.15499300	-1.06145400	0.99997400
C	-5.56644900	-2.28161100	1.51022400
C	-3.38481700	0.33340200	-0.12641100
C	-2.16463900	0.36138600	-0.76250000
C	-1.36822400	-0.85761900	-0.84858800
C	-0.20735600	-0.98992400	-1.54801400
C	-1.68369000	1.65064600	-1.29620700
H	-2.76901300	-4.11884500	0.77297400
H	-5.82933400	-0.20421300	1.04817700
H	-6.55621700	-2.37250100	1.95553300
H	-2.36520000	2.50157000	-1.05837900
C	-4.28156900	1.52180700	-0.06551100

C	-4.40334100	2.33303700	1.07913500
C	-5.08871900	1.79280900	-1.17460600
C	-5.31115800	3.39872100	1.08291000
C	-5.99026900	2.85409500	-1.16018200
H	-5.00014700	1.15822900	-2.05923200
C	-6.10293000	3.66052900	-0.02935000
H	-5.38261800	4.02222100	1.97470000
H	-6.60661800	3.05128200	-2.04027200
H	-6.80645700	4.49547300	-0.01481700
C	-3.54785200	2.06709700	2.26687700
O	-2.72523300	1.18950800	2.33898900
O	-3.78149800	2.91462200	3.27610800
H	-3.17447500	2.66345900	3.98876300
O	-1.81602000	-1.93761600	-0.16175100
O	-5.01521300	-4.60983400	1.89369600
C	-6.27511800	-4.83572500	2.47677200
H	-7.08891500	-4.61536000	1.76349000
H	-6.42088000	-4.20031100	3.36818000
C	-6.32943700	-6.29199100	2.87596200
H	-5.50400000	-6.51360800	3.58192400
H	-6.17118900	-6.92713300	1.98135100
O	-7.57638900	-6.51607700	3.45365700
C	-7.77947600	-7.83295300	3.87503300
H	-7.02771100	-8.13532700	4.63114400
H	-7.69532900	-8.54472100	3.02971000
C	-9.16276300	-7.92681600	4.47552700
H	-9.91385700	-7.62112000	3.71899300
H	-9.24654400	-7.21334800	5.32044400
O	-9.36166200	-9.24094300	4.89300900
C	-10.61110000	-9.46974600	5.47220100
H	-11.43360900	-9.23901800	4.76521000
H	-10.76553700	-8.83302000	6.36681500
C	-10.69099100	-10.92409900	5.87419300
H	-10.52750600	-11.54592500	4.97184900
H	-9.85880100	-11.14005700	6.57309100
O	-11.94932500	-11.13173000	6.45232200
H	-12.01663900	-12.05387300	6.71353400
O	-0.67069100	1.84522000	-1.92453800
C	0.62369600	-2.17031100	-1.68535800
O	1.64702400	-2.13503700	-2.35995200
C	0.26909500	-3.54595600	-1.06485300
H	0.03112500	-3.44342300	0.00529200
O	1.34564600	-4.37574600	-1.25488500
H	1.92902200	-3.86288200	-1.85319300

O	−0.90147400	−4.04039100	−1.66472800
H	−0.64502600	−4.33425500	−2.54773600
H	0.15655600	−0.10225700	−2.06170400

FTEG

C	−4.71219600	−3.42296600	1.35801500
C	−3.46182800	−3.28223300	0.75105900
C	−3.06740400	−2.03971700	0.27695600
C	−3.90092900	−0.90673300	0.39082100
C	−5.15251300	−1.07954500	1.00132500
C	−5.56589800	−2.31114000	1.48224400
C	−3.41519800	0.34960500	−0.11497000
C	−2.18151800	0.40583000	−0.71543800
C	−1.37299800	−0.79463700	−0.80559900
C	−0.14308600	−0.80565800	−1.37987700
H	0.43468800	−1.72907300	−1.42978200
C	0.44313900	0.40653900	−1.93856100
C	−0.38529300	1.62377700	−1.82903000
C	−1.60882700	1.61596000	−1.25636400
H	−2.79980100	−4.14216200	0.65283600
H	−5.81337100	−0.21570800	1.09774400
H	−6.54460400	−2.40551500	1.95084100
H	0.05136600	2.53744100	−2.23862200
H	−2.19879800	2.53271800	−1.18676400
C	−4.29281200	1.54730500	−0.04772600
C	−4.38218000	2.37634000	1.08747900
C	−5.07066300	1.85355100	−1.16867600
C	−5.23678300	3.48571000	1.07312500
C	−5.92082000	2.95662000	−1.17192400
H	−4.99854400	1.21151400	−2.04932100
C	−6.00567800	3.77577900	−0.04795700
H	−5.28588600	4.12015100	1.95861800
H	−6.51755100	3.17719700	−2.06005400
H	−6.66873300	4.64344800	−0.04589900
C	−3.56037000	2.07966500	2.29268600
O	−2.83742800	1.12478100	2.41985800
O	−3.70473300	2.99913600	3.25683200
H	−3.13276100	2.71662100	3.98635300
O	1.54807500	0.43123000	−2.46582000
O	−1.85537900	−1.96019500	−0.30025000
O	−5.02058200	−4.65463400	1.79230500
C	−6.25823500	−4.88036000	2.41891200
H	−7.09790900	−4.64346200	1.74147200
H	−6.36618600	−4.25811100	3.32504600

C	−6. 30880600	−6. 34221200	2. 79769500
H	−5. 46059500	−6. 57978000	3. 47081000
H	−6. 18629400	−6. 96467100	1. 88852100
O	−7. 53588300	−6. 56925600	3. 41567900
C	−7. 72844100	−7. 88973500	3. 82941300
H	−6. 95277200	−8. 20227700	4. 55683100
H	−7. 67459100	−8. 59286800	2. 97431400
C	−9. 09106400	−7. 98682300	4. 47496300
H	−9. 86653400	−7. 67331100	3. 74679800
H	−9. 14507900	−7. 28070800	5. 32839800
O	−9. 27847000	−9. 30435900	4. 88749000
C	−10. 50888500	−9. 53630000	5. 50481300
H	−11. 35346300	−9. 30038300	4. 82611200
H	−10. 63415600	−8. 90550700	6. 40813900
C	−10. 57699300	−10. 99327300	5. 89942900
H	−10. 44197900	−11. 60910100	4. 98828000
H	−9. 72354100	−11. 21414800	6. 57061500
O	−11. 81679500	−11. 20452700	6. 51506700
H	−11. 87588400	−12. 12813100	6. 77306800

TDDFT output files

fhztddft.log fobatddft.log ftegtddft.log

(Dr Min Feng from Nankai University was appreciated for using ChemDraw and Gaussian to make above figure and data respectively)
